# Abdominal Near Infrared Spectroscopy can be reliably used to measure splanchnic oxygenation changes in preterm infants

**DOI:** 10.1038/s41372-022-01576-2

**Published:** 2022-12-10

**Authors:** R. A. Thomas, M-R Ballard, N. Aladangady, J. Banerjee

**Affiliations:** 1 Neonatal unit, Queen Charlotte’s and Chelsea Hospital, Imperial College Healthcare NHS Trust, London, UK; 2 Neonatal unit, Homerton Healthcare NHS Foundation Trust, London, UK; 3 Centre for Genomics and Child Health, Barts and The London School of Medicine and Dentistry, Queen Mary University of London, London, UK; 4 Biomedical Research Centre, Imperial College Healthcare NHS Trust, London, UK; 5 Institute of Reproductive and Developmental Biology, Imperial College London, London, UK; 6 Origins of Health and Disease, Centre for Child Health, Imperial College London, London, UK

**Keywords:** Physical examination, Predictive markers

## Abstract

**Objective:**

Near-infrared spectroscopy (NIRS) allows assessment of regional tissue oxygen delivery and extraction. There are doubts regarding reliability of gut NIRS measurements. This study assesses reliability of NIRS for monitoring gut oxygenation.

**Study design:**

Splanchnic tissue haemoglobin index (sTHI), tissue oxygenation index (sTOI) and fractional tissue oxygen extraction (sFTOE) changes during blood transfusion were measured using NIRS and compared to stable control infants. Infants were grouped into 3 chronological age groups: 1–7, 8–28 and ≥29 days of life.

**Results:**

sTHI, sTOI significantly increased, and sFTOE reduced following blood transfusion in all age group infants (*n* = 59), with no changes seen in control infants (*n* = 12). Baseline characteristics including gestational age and feed volumes did not differ between groups.

**Conclusion:**

Gut perfusion measured by NIRS improved in infants who received blood transfusion, a change not seen in the control group, thus suggesting NIRS is a reliable method to measure splanchnic tissue oxygenation.

## Introduction

First described in 1977, the use of NIRS in neonatology has significantly expanded over recent years [1]. Whilst pulse oximetry measures pulsatile blood flow and oxygenated hemoglobin in arterial circulation, NIRS detects both oxygenated (O_2_Hb), and deoxygenated hemoglobin (HHb) in the tissue of interest, from a combination of veins, arteries and capillaries reflecting true tissue oxygenation [2]. The difference between O_2_Hb and HHb is calculated to reflect tissue oxygenation, and reported as regional tissue oxygenation (rSO_2_) or tissue oxygenation index (TOI) [3]. NIRS detects both oxygenated (O_2_Hb) and deoxygenated hemoglobin (HHb) through veins, arteries and capillaries/arterioles in a ratio of 75:20:5 respectively [4] and hence closely reflects venous oxygenation levels. Efforts to validate it have used various measurements of venous oxygenation and early studies comparing cerebral NIRS and jugular venous oxygen saturation show close correlation [4]. The use of NIRS has since expanded and includes assessment of regional tissue saturation elsewhere [5]. Abdominal or splanchnic NIRS as a surrogate for monitoring gut perfusion in the neonatal population has been widely studied with literature supporting both the safety and feasibility of continuous monitoring [6, 7], however, its reliability in clinical practice is debated [2].

This prospective observational study aimed to investigate the reliability of abdominal or splanchnic tissue oxygenation measured by NIRS in preterm infants.

## Methodology

Splanchnic regional oxygen saturation monitoring was prospectively carried out on preterm infants admitted to the neonatal unit. The abdominal or splanchnic regional NIRS measurements of preterm infants who received blood transfusions were compared with a control group of stable preterm infants who did not receive transfusions. Any preterm baby due to receive a blood transfusion was deemed eligible and consent sought from parents prior to inclusion. Babies who were moribund or those affected by life-limiting congenital anomalies were excluded. Additionally, babies were excluded if they received transfusion at a time when parents were not present for consent or the research team was not available to perform measurements. In the blood transfusion group, the infants were recruited to three groups based on chronological age at the time of blood transfusion and regional saturations monitoring. Group 1 included infants who received transfusion in the 1–7 days of life, group 2 included those transfused at 8–28 days of life, and group 3 included those ≥29 days of life. The groups were chosen as the haemoglobin threshold for blood transfusions differ in those postnatal age groups in the hospital protocol. This was in liaison with the British Transfusion Society (BTS) guidance [8]. RBCs transfused were plasma depleted packed cell, CMV negative, irradiated blood stored in routine storage solution as recommended by the NHS Blood and Transplant organisation (www.nhsbt.nhs.uk). Infant characteristics were collected as follows: gestational age at delivery (completed weeks), birth weight (g), chronological age (days) and weight at transfusion (g), haemoglobin at birth (g/dL), pre-transfusion haemoglobin (g/dL), total fluids (ml/kg/day) and total enteral feeds (ml/kg/day).

Infants receiving packed red cell transfusions did so at 15 ml/kg administered over a period of 3 hours. Decisions regarding indication for transfusion varied and were made as per local unit guidance which was in line with the NHS BTS guidelines. Hb thresholds used for transfusion were ≤13 g/dl, ≤10 g/dl and ≤7.5 g/dl for stable babies within the first week of life, ≥8 to 28 days of life and ≥29 days of life respectively. In addition, cardio-respiratory support was taken into consideration in the decision made to transfuse. Enteral feeding was not held during the transfusion and milk feed choice was as per local unit guidance (maternal or donor expressed breast milk, or preterm formula).

Splanchnic regional oxygen saturations were performed using the NIRS device NIRO 300, Hamamatsu Photonics KK, Japan. Parameters recorded were splanchnic tissue oxygenation index (sTOI), tissue hemoglobin index (sTHI), and fractional tissue oxygen extraction (sFTOE). sFTOE was calculated using measured SaO_2_, and sTOI using the formula [9]:

$$sFTOE = 100 \times \left( {\frac{{SaO_2 - sTOI}}{{SaO_2}}} \right)$$

The NIRO 300 NIRS probe was placed in the infraumbilical region in all infants and was held in place with a non-constricting light impervious band. Probe placement was standardized for all infants and saturations data was evaluated for motion artefacts. For those who received transfusion the NIRS measurements were started 15–20 min prior to transfusion and kept in place for the three hours (5 ml/kg/h) of transfusion; this was discontinued 15–20 minutes post-transfusion. For the infants in the control group the NIRS probe was kept for a period of 3 hours.

A mean for 15-minute epochs of NIRS oximetry measurements were determined for each infant using mathematical software MATLAB (Math works, Natick, MA, USA) during the following time periods: T1 – 15–20 min before the start of the blood transfusion, T2 – 1 h into blood transfusion, T3 –2 h into blood transfusion and T4 – 15–20 min post-blood transfusion. The mean of these epochs was then compared using repeated-measures ANOVA with Bonferroni correction. Regional saturation data were evaluated for motion artefacts using MATLAB. The pre- and post-transfusion values of all measurements were compared using paired (two-tailed) t-test. A *P-*value of <0.05 was considered significant. These measurement changes were then compared with the three-hour values of splanchnic NIRS monitoring in the control cohort. The data were analysed using SPSS 22.0 software (IBM Corp., North Castle, NY, USA). Parental written consent was obtained. The study was approved by the National Research Ethics Committee (NREC) (REC no.12/LO/0527) and was adopted as an NIHR portfolio study (NIHR Study ID 13594).

## Results

The study included 71 infants of which seven were later excluded from analysis due to motion artefacts. All seven excluded infants were from the transfused group (three from group 1 (1–7 days of life), one from group 2 (8–28 days of life), and three from group 3 (≥29 days of life)). Gestational age at birth ranged from 25 to 27 weeks. The characteristics for infants in the transfused groups (*n* = 59) and the control group (*n* = 12) at birth are demonstrated in Table 1, and during the study period are shown in Table 2. Of the twelve infants in the control group, four were studied in the first week of life, five between day 8 to day 28 of life and three were ≥29 days of postnatal age. The mean gestational age at birth was 29 ± 5 weeks and birth weight was 1400 ± 972 grams. The mean postnatal age at measurement was 20 ± 15 days. The mean haemoglobin (Hb) level at birth was 13.3 ± 2.8 mg/dl and the pre-measurement Hb was 10.3 ± 2.8 mg/dl. Two infants were undergoing invasive ventilation, five each were undergoing non-invasive ventilation or breathing in air. None of the babies were on inotropic support or receiving treatment for suspected or proven sepsis. Eight infants had no IVH and four had Grade 1 haemorrhage.

**Table 1:** Infant characteristics at birth for all groups.

Characteristics	Group 1 (1–7 days) *N* = 20	Group 2 (8–28 days) *N* = 21	Group 3 (≥29 days) *N* = 18	Control group *N* = 12
Gestational age (completed weeks)	26 (23–27)	25 (23–30)	26 (24–34)	27 (24–33)
Birth weight (grams)	763 (600–1180)	740 (600–1240)	793 (520–1746)	804 (528–2372)
Haemoglobin at birth (g/dl)	14.5 (9.8–20.7)	14.7 (10.0–17.4)	15.3 (10–18.9)	13.3 (10.5–16.1)
Maternal PET	3 (15)	5 (24)	4 (22)	2 (17)
IUGR	3 (15)	5 (24)	4 (22)	3 (25)
Chorioamnionitis	9 (45)	8 (38)	8 (44)	5 (42)
Antepartum haemorrhage	6 (30)	8 (38)	4 (22)	4 (33)
Antenatal steroids	17 (85)	20 (95)	16 (89)	10 (83)

**Table 2:** Infant characteristics on the day of NIRS measurements.

Characteristics Median (range)	Group 1 (0–7 days) *N* = 20	Group 2 (8–28 days) *N* = 21	Group 3 (>28 days) *N* = 18	Control group *N* = 12
Chronological age (days)	5 (1–7)	14 (8–27)	45 (29–93)	14 (8–42)
Total fluids (ml/kg/d)	150 (90–180)	150 (100–180)	165 (100–180)	150 (120–180)
Total feeds (ml/kg/day)	18 (0–70)	120 (0–180)	155 (0–180)	90 (60–180)

Further details regarding the transfused infant groups at the time of transfusion are described in Table 3.

**Table 3:** Transfused infant characteristics at red cell transfusion.

Characteristics	Group 1 (1–7 ds) *n* = 20	Group 2 (8–28 ds) *n* = 21	Group 3 (≥29 ds) *n* = 18
Chronological age (days)	5 (1–7)	14 (8–27)	45 (29–93)
Weight at transfusion (grams)	774 (700–1180)	805 (680–1250)	1125 (887–2045)
Invasive/Non-invasive ventilation/nasal cannula oxygen or breathing in air	13 (65)/7(35)/0 (0)	13 (62)/7 (33)/1 (5)	6 (33)/9 (50)/3 (17)
Presence of PDA	19 (95)	12 (57)	1 (6)
Presumed sepsis on antibiotics	19 (95)	8 (38)	8 (44)
Pre-transfusion Hb (g/dl)	11.0 (8.5–13.1)	10.3 (7.7–12.2)	9.2 (7–10.9)

**Fig. 1:**
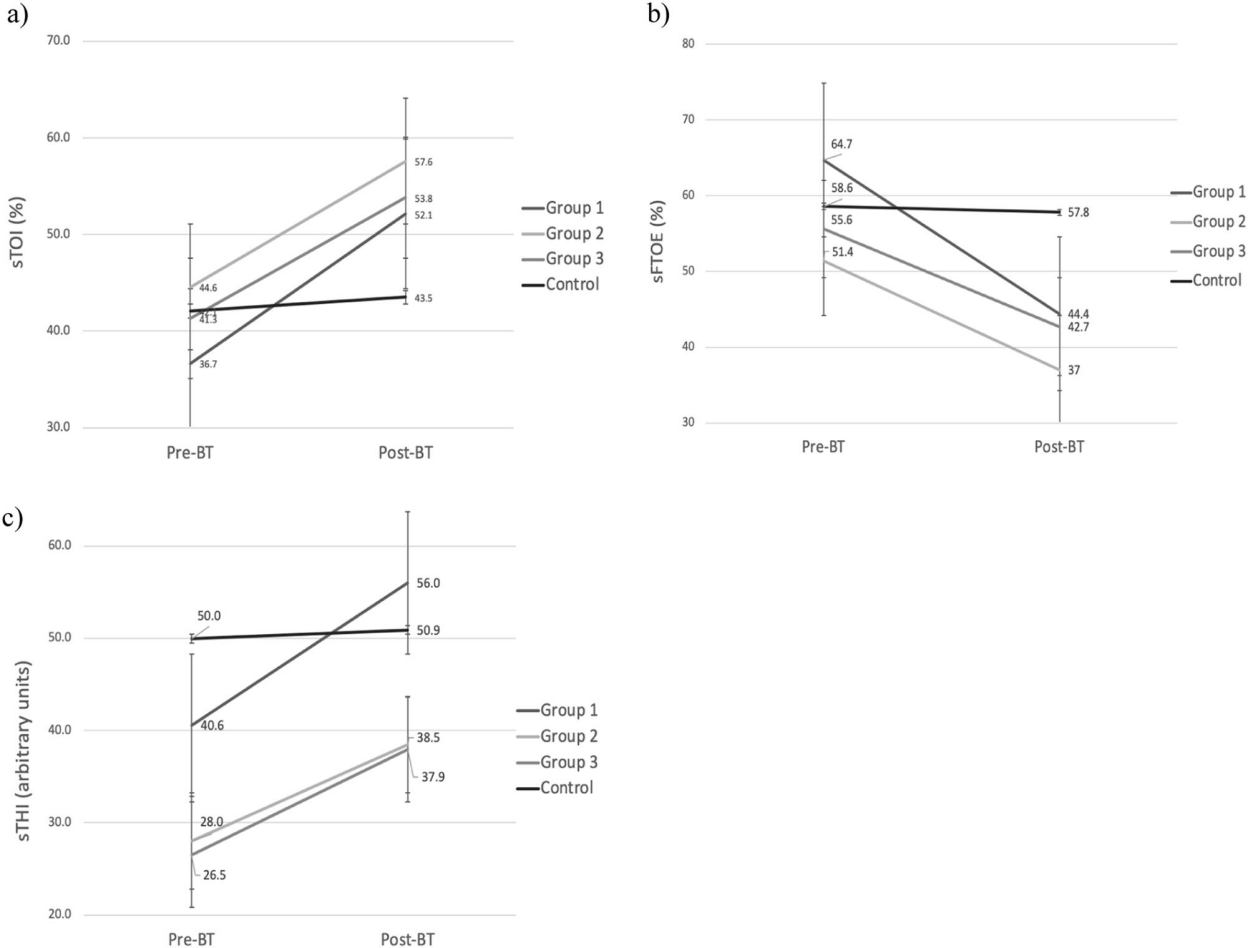
Splanchnic NIRS measurements in transfused and control preterm infant groups. Changes in sTOI (**a**), sFTOE (**b**) and sTHI (**c**) in transfused and control groups. **p < 0.001, *p < 0.01, NS not significant.

In the control group infants, sTHI, sTOI, sFTOE remained unchanged over the period of 3 hours of NIRS monitoring (Fig. 1). In infants who received blood transfusion, sTHI and sTOI increased significantly across all groups following transfusion (*p* < 0.05) (Table 4). The sTHI increased by 39% (*p* = 0.001), 45% (*p* = 0.001), and 47% (*p* = 0.001) in groups 1, 2, and 3 respectively. Baseline sTOI increased by 42% (*p* = 0.01), 29% (*p* = 0.01), and 30% (*p* = 0.01) in groups 1, 2, and 3. Pre-transfusion sFTOE decreased significantly by 31% (*p* = 0.004), 28% (*p* = 0.005), and 23% (*p* = 0.0004) in groups 1, 2, and 3 respectively following blood transfusion (Table 4).

**Table 4:** Splanchnic NIRS measurement parameters in transfused and control preterm infants.

Splanchnic oximetry parameters Mean (SD)	Group 1 (1–7 days) *n* = 17^†^			Group 2 (8–28 days) *n* = 20^††^			Group 3 (≥29 days) *n* *=* 15^†††^			Control infants *n* *=* 12		
	Pre-BT	Post-BT	*p* value	Pre-BT	Post-BT	*p* value	Pre-BT	Post-BT	*p* value	Initial measurement	Measurement after 3 hours	*p* value
Splanchnic tissue haemoglobin index (sTHI) (percentage increase from baseline) %	40.6 (15.4)	56.0 (14.8)	0.001	37.0 (12.5)	53.6 (16.8)	0.001	26.6 (12.2)	37.9 (14.8)	0.001	38.9 (14.6)	40.5 (16.2)	0.74
Splanchnic tissue oxygenation index (sTOI) %	36.7 (19.3)	52.1 (20.8)	0.01	44.6 (10.4)	57.6 (14.3)	0.01	41.3 (10.4)	53.8 (16.5)	0.01	42.1 (10.4)	43.5 (11.3)	0.67
Splanchnic fractional tissue oxygen extraction (sFTOE)%	64.7 (13.4)	44.4 (20.3)	0.004	51.4 (11.5)	37.0 (14.9)	0.005	55.6 (11.8)	42.7 (15.1)	0.0004	58.6 (12.5)	57.8 (13.4)	0.78

*n number of infants studied.*

^*†*^ *3 infants*, ^*††*^ *1 infant, and* ^*†††*^ *3 infants excluded from the analysis due to motion artefacts.*

*Pre-BT– pre-blood transfusion.*

*Post-BT–Post-blood transfusion.*

## Discussion

The current study demonstrated that gut oxygenation parameters measured by NIRS significantly increased following blood transfusion whereas the gut perfusion remained stable over the same time in non-transfused controls, thereby confirming the clinical validity of gut oxygenation measurement using NIRS. Abdominal or splanchnic tissue oxygenation measurements using NIRS has been extensively studied as a marker of gut perfusion in the preterm neonatal population. Gay et al. in a recent study investigated abdominal NIRS of 29 premature piglets [10]. Three of the piglets developed necrotizing enterocolitis (NEC); this group of piglets had significantly lower abdominal NIRS values than the control group. They concluded that splanchnic NIRS can detect reduced intestinal perfusion and subsequently predict the risk of NEC. These findings demonstrate that splanchnic NIRS has a place in neonatal care however interpretation remains unclear due to a lack of established threshold and normal values.

In the present study splanchnic tissue oxygenation (sTOI), haemoglobin index (sTHI), and fractional tissue oxygenation extraction (FTOE) were compared at a range of chronological ages in preterm infants who received packed red cell transfusion versus those who did not. Splanchnic tissue oxygenation markers such as sTOI and sTHI increased in infants following packed red cell transfusion across all chronological age groups studied. In comparison, these parameters remained stable without significant changes in control infants. Similar findings have been demonstrated in a smaller study by Mintzer et al. [11], in which blood transfusion in 10 neonates resulted in an increase in regional tissue oxygenation in the cerebral, splanchnic, and renal circulation. Other studies (Dani et al. [12], Bailey et al. [13]) have demonstrated similar increases in cranial and splanchnic oxygenation following packed red cell transfusion. But this study was the first to compare the splanchnic tissue oxygenation measurements with a comparator group who received no transfusions.

There was a statistically significant reduction in fractional tissue oxygen extraction (FTOE) in transfused infants in all 3 groups. The FTOE ratio provides an estimate of oxygen delivery and utilization, in keeping with an expected adaptive tissue response following the increased oxygen delivery. Schat et al. examined liver, cerebral and infraumbilical regional saturation and FTOE and showed the use of FTOE useful to identify NEC and complicated NEC (Bell’s stage 3B or death) [14]. Mintzer et al. found reduction in FTOE in splanchnic, renal and cerebral circulation following transfusion and suggested FTOE may be a more sensitive marker of anemia in neonates [11]. Several other studies support this including an observational study by Santal et al. [15] although others reported a lack of correlation of FTOE and anaemia [16].

In this study, enteral feeds were continued in both the control and transfused group. The timing of enteral feeds in relation to the NIRS monitoring was not within the scope of this study but will be of interest in future research. Comparing splanchnic and cerebral oxygenation (SCOR) has been widely studied as a potential marker for differential cerebral and splanchnic tissue oxygenation noted in symptomatic anaemia. Previously, studies have used splanchnic NIRS to assess gut tissue perfusion during enteral feeding, with changes in splanchnic blood flow and SCOR during bolus feeding demonstrated [17, 18]. A significant reduction in SCOR has been demonstrated in anaemic infants following enteral feeds [19]. In preterm infants with abnormal antenatal doppler studies, lower baseline gut tissue saturations were found to be associated with development of abdominal complications including necrotising enterocolitis and feed intolerance [20].

Anaemia of prematurity is a common complication in preterm infants [21], with 90% of extremely low birth weight infants receiving packed red blood cell transfusions. Blood transfusions have well-established risks and have been associated with complications of prematurity including intraventricular haemorrhage, retinopathy of prematurity, chronic lung disease, and a temporal association with necrotizing enterocolitis [22–24]. The timing, volume administered, and threshold (haemoglobin level and clinical signs) for packed red cell transfusions vary widely across neonatal units with optimal management still debated [25–27]. Clinical practice is moving toward preventative strategies such as minimal blood taking, increasing use of point of care tests, transcutaneous CO_2_ monitoring to reduce capillary blood gases, and use of cord blood for transfusions [28]. Helping to identify infants with symptomatic anaemia would therefore be of great benefit to aid decision-making regarding transfusion threshold at the cot-side. Early identification of symptomatic infants as opposed to the use of set thresholds could reduce the risk of unnecessary transfusions and associated risks. Herein lies the potential role for NIRS; by measuring splanchnic tissue perfusion and thus demonstrating compromised oxygen delivery in anemic states which could then guide timing of blood transfusion.

Abdominal or splanchnic NIRS does pose several challenges and is influenced by variables including tubular intestinal structure, gaseous distension, peristalsis, the presence of faecal matter and meconium, and a larger surface area which have potential to influence data [29]. Physiological variations in splanchnic saturations over the first 3 weeks of life in stable preterm babies have been demonstrated by several groups, with a gestational age-dependent nadir towards the end of the first week of life [2, 30]. It is these multiple variables and a lack of validated normal ranges for parameters measured by NIRS which have led to debate regarding its reliability however our study does demonstrate that changes in NIRS parameters were reliably measurable across range of gestational ages and postnatal ages in preterm infants. This suggests that NIRS can be used reliably as a marker for gut tissue perfusion. Furthermore, the lack of significant change in sTOI, sTHI, and sFTOE in the control group over time supports this further.

There are some limitations to this study. The population size is relatively small with a discrepancy between the control group (*n* = 12) and transfused group (*n* = 59). Although there was spread across various gestational age and postnatal age in the transfused group of infants, 7 infants were excluded due to motion artefacts. Transfusion was carried out as per the unit policy with regards to hemoglobin thresholds and volume of blood administered (15 ml/kg) over 3 hours, which is likely to differ from other neonatal units given the wide variation in practice [24, 31]. The device used in this study was the NIRO 300 (Hamamatsu Photonics KK, Japan). Given the wide range of devices available globally will inevitably lead to variation in measurements and therefore may impact on reliability. NIRS does, as described previously, have inherent limitations in that it is a surrogate marker for gut perfusion and potentially influenced by several factors including interference from bowel loops and gas and motion artefacts. Additionally, placing NIRS probes on infra-umbilical area of extremely preterm infants could be challenging, although this was not noted to cause any issues in the present study. An important consideration when interpreting NIRS data is the type of sensor and device used (INVOS-5100 and NIRO-300 being the two most used devices) as there are significant differences in regional oxygenation measurements between different recording devices [32]. Hence NIRS should be used to monitor trends rather than absolute values.

## Conclusion

This study reports significant changes in splanchnic NIRS, as a surrogate marker for splanchnic tissue perfusion, in infants during and post blood transfusion. There was an improvement in sTOI and sTHI, with reduced FTOE following blood transfusion in a wide range of gestational age and postnatal age group of preterm infants. No changes in NIRS measurements were seen over the same duration in similar infants who did not receive a blood transfusion. This, therefore, suggests that the measurements are a true reflection of changes in splanchnic tissue oxygenation during blood transfusion, and that NIRS could be a reliable technique to monitor gut perfusion in preterm neonates.

## Data Availability

The data that support the findings of this study are available from the corresponding author, JB, upon reasonable request.
